# Sodium thiosulfate, a source of hydrogen sulfide, stimulates endothelial cell proliferation and neovascularization

**DOI:** 10.3389/fcvm.2022.965965

**Published:** 2022-10-03

**Authors:** Diane Macabrey, Jaroslava Joniová, Quentin Gasser, Clémence Bechelli, Alban Longchamp, Severine Urfer, Martine Lambelet, Chun-Yu Fu, Guenter Schwarz, Georges Wagnières, Sébastien Déglise, Florent Allagnat

**Affiliations:** ^1^Department of Vascular Surgery, Lausanne University Hospital, Lausanne, Switzerland; ^2^Laboratory for Functional and Metabolic Imaging, LIFMET, Swiss Federal Institute of Technology (EPFL), Lausanne, Switzerland; ^3^Institute of Biochemistry, Department of Chemistry & Center for Molecular Medicine, Cologne University, Cologne, Germany

**Keywords:** angiogenesis, hydrogen sulfide, arteriogenesis, thiosulfate, peripheral arterial disease, endothelial cells, inflammation

## Abstract

Therapies to accelerate vascular repair are currently lacking. Pre-clinical studies suggest that hydrogen sulfide (H_2_S), an endogenous gasotransmitter, promotes angiogenesis. Here, we hypothesized that sodium thiosulfate (STS), a clinically relevant source of H_2_S, would stimulate angiogenesis and vascular repair. STS stimulated neovascularization in WT and LDLR receptor knockout mice following hindlimb ischemia as evidenced by increased leg perfusion assessed by laser Doppler imaging, and capillary density in the gastrocnemius muscle. STS also promoted VEGF-dependent angiogenesis in matrigel plugs *in vivo* and in the chorioallantoic membrane of chick embryos. *In vitro*, STS and NaHS stimulated human umbilical vein endothelial cell (HUVEC) migration and proliferation. Seahorse experiments further revealed that STS inhibited mitochondrial respiration and promoted glycolysis in HUVEC. The effect of STS on migration and proliferation was glycolysis-dependent. STS probably acts through metabolic reprogramming of endothelial cells toward a more proliferative glycolytic state. These findings may hold broad clinical implications for patients suffering from vascular occlusive diseases.

## Introduction

The prevalence of peripheral artery disease (PAD) is constantly rising as the prevalence of aging, hypertension, and diabetes mellitus (1, 2). PAD is mostly due to atherosclerosis, which leads to progressive obstructions of peripheral arteries. When pharmacological therapy (lipid-lowering (3) and antihypertensive drugs (4) and life-style changes [diet, exercise…(5)] fail and PAD progresses to critical limb threatening ischemia (CLTI), vascular surgery remains the only option (1, 2). However, surgery may fail to relieve symptoms, or may not be possible due to the anatomy, severity of the disease or comorbidities. Thus, 20–40% of CLTI patients are not amenable to revascularization or have failed revascularization (6). Even in the case of a successful surgery, residual microvascular disease may remain. New strategies to promote neovascularization and recovery following surgical revascularization in PAD and CLTI patients are required.

Hydrogen sulfide (H_2_S) is a gasotransmitter produced in mammals via the reverse transsulfuration pathway (7). H_2_S is now recognized as having important vasorelaxant, cytoprotective, and anti-inflammatory properties (7). Moreover, pre-clinical *in vivo* studies showed that H_2_S donors (NaHS, GYY4137) promote reperfusion in mice after femoral artery ligation in a model of hindlimb ischemia (8, 9). Rushing et al. further showed that SG1002, a H_2_S releasing pro-drug, increases leg neovascularization and collateral vessel formation after occlusion of the external iliac artery in miniswine (10). In these studies, H_2_S improved angiogenesis and arteriogenesis, two processes central for ischemic skeletal muscle repair (11, 12). Overall, H_2_S stimulates EC proliferation, migration and angiogenesis in a variety of pre-clinical models (13–15).

Mechanistically, H_2_S stimulates the persulfidation of the VEGF receptor VEGFR2, promoting dimerization, auto phosphorylation and activation in EC (16). H_2_S also promotes eNOS activity and NO production, which is instrumental to angiogenesis (7). In addition, H_2_S inhibits mitochondrial oxidative phosphorylation, which in EC specifically, increases glycolytic adenosine triphosphate (ATP) production to provide rapid energy for EC proliferation and migration (17). However, despite these potent cardiovascular benefits in pre-clinical studies, H_2_S-based therapeutics are not available yet.

Sodium thiosulfate (Na_2_S_2_O_3_) is clinically-approved for the treatment of cyanide poisoning (18) and calciphylaxis, a rare condition of vascular calcification affecting patients with end-stage renal disease (19). Sodium thiosulfate (STS) participates in sulfur metabolism within cells, releasing H_2_S through non-enzymatic and enzymatic mechanisms (20, 21). STS protects rat hearts from ischemia reperfusion injury (22), and we recently demonstrated that STS reduces intimal hyperplasia in pre-clinical models (23).

The aim of this study was to test whether STS stimulates arteriogenesis and angiogenesis in a mouse model of hindlimb ischemia (HLI). STS promoted vascular recovery following ischemia in WT and hypercholesterolemic LDLR^–/–^ mice. STS also promoted VEGF-dependent angiogenesis *in vivo* in a matrigel plug assay and *in ovo* in the Chick Chorioallantoic Membrane (CAM) angiogenesis assay. As expected, STS promoted HUVEC proliferation and migration, similarly to other H_2_S donors. Finally, STS inhibited mitochondrial respiration and promoted glycolysis in EC, and inhibition of glycolysis abrogated the effect of STS in HUVEC.

## Materials and methods

### Mice

WT mice C57BL/6JRj mice were purchased form Janvier Labs (Le Genest-Saint-Isle, France). LDLR^–/–^ mice (24) (*Ldl*^*rTM*1Her^, JAX stock #002207, kindly provided by Prof. Caroline Pot, Lausanne university Hospital, Switzerland) were bred and housed in our animal facility and genotyped as previously described (24). All mice were housed at standard housing conditions (22°C, 12 h light/dark cycle), with *ad libitum* access to water and regular diet (SAFE^®^ 150 SP-25 vegetal diet, SAFE diets, Augy, France). LDLR^–/–^ mice were put on a cholesterol rich diet (Western 1635, 0.2% Cholesterol, 21% Butter, U8958 Version 35, SAFE^®^ Complete Care Competence) for 3 weeks prior to surgery. Mice were randomly treated with sodium thiosulfate (STS). Sodium Thiosulfate (Hänseler AG, Herisau, Switzerland) was given in mice water bottle at 2 or 4 g/L to achieve 0.5 or 1 g/kg/day, changed three times a week.

Hindlimb ischemia surgery was performed under isoflurane anesthesia (2.5% under 2.5 L O_2_). Local anesthesia was ensured by subcutaneous injection with a mix of lidocaine (6 mg/kg) and bupivacaine (2.5 mg/kg) along the incision line. The femoral artery was exposed through a small incision in the upper part of the leg. Two sutures (7-0 silk) were placed above the bifurcation with the epigastric artery. The femoral artery was then cut between the two sutures and the incision was closed with 5-0 prolene. Buprenorphine (0.1 mg/kg Temgesic, Reckitt Benckiser AG, Switzerland) was provided before surgery, as well as a post-operative analgesic every 12 h for 36 h. Perfusion of both operated and non-operated contralateral leg was monitored using a High Resolution Laser Doppler Imager (moorLDI2-HIR; Moor Instruments) under isoflurane anesthesia on a heating pad. Mice were euthanized under anesthesia by cervical dislocation and exsanguination 2 weeks post-surgery. Muscles were either frozen in OCT for histology, or flash frozen directly in liquid nitrogen for molecular analyses.

Matrigel plug assay was conducted using Growth factor reduced Matrigel (BD Biosciences) supplemented with 20 U/ml heparin (L6510, Seromed) and 200 ng/ml human VEGF 165 (100-20, Peprotech), supplemented or not with 15 mM STS. Under isoflurane anesthesia (2.5% under 2.5 L O_2_), 400–500 μl of Matrigel was injected subcutaneously on the back of the mouse with a 25G needle. Matrigel plugs were isolated 7 days after implantation, dissolved overnight in 0.1% Brij L23 (Sigma-Aldrich). Hemoglobin content was measured in a 96 well plate via colorimetric assay using 20 μl of samples and 180 μl Drabkin’s reagent (D5941, Sigma-Aldrich). Absorbance was measured after 20 min incubation at RT in the dark at 540 nM using a Synergy Mx plate reader (BioTek Instruments AG, Switzerland). Data were plotted against a standard curve of ferrous stabilized human Hemoglobin A0 (H0267, Sigma-Aldrich).

EdU (A10044, ThermoFischer Scientific) was diluted in NaCl at a concentration of 2 mg/ml and 500 μg was injected via i.p. injection 16 h before sacrifice. Mice were sacrificed at day 4 post HLI; ischemic muscles were placed in OCT and frozen in liquid nitrogen vapor.

All animal experimentations conformed to *the National Research Council:* Guide for the Care and Use of Laboratory Animals (25). The Cantonal Veterinary Office (SCAV-EXPANIM, authorization number 3504) approved all animal care, surgery, and euthanasia procedures.

### Cell culture

Pooled human umbilical vein endothelial cells (HUVECs; Lonza) were maintained in EGM™-2 (Endothelial Cell Growth Medium-2 BulletKit™; Lonza) at 37°C, 5% CO_2_ and 5% O_2_ as previously described (26). Passages 1–8 were used for the experiments.

### Chicken chorioallantoic membrane

Fertilized brown chicken eggs were purchased from Animalco AG, Switzerland. Eggs were incubated for 3 days at 37°C in a rotating incubator with the blunt end up. A small 3 mm diameter hole was made at embryo development day (EDD) 3. The Hole was covered with tape and the eggs placed back in the incubator in a stationary position. On EDD 11, the hole was enlarged to a diameter 25 mm, enabling topical administration of 20 μl of a fresh STS solution [500 μM–20 mM in sodium chloride solution (NaCl; BioConcept, Switzerland)]. On the control eggs, 20 μl of 0.9% NaCl was added. The concentrations of STS are relative to the weight of the embryo at EDD 11. After STS administration, the hole was covered with a parafilm and eggs were returned to the incubator. At EDD 13, 20 μl of fluorescein isothiocyanate – dextran (25 kDa, 25 mg/ml; Sigma–Aldrich, Switzerland) dissolved in NaCl was intravenously injected to the CAM’s vascular network. At the same time, 100 μl of India ink (Parker) was injected under the CAM to improve the contrast between the blood vessels and extravascular space. It should be noted that India ink in this quantity and these conditions is not toxic to the CAM (27). An epifluorescent microscope (Eclipse E 600 FN Nikon) was used for the acquisition of the angiograms using a 10× objective (Nikon, Plan Fluor, NA: 0.30, WD 16.0). Effect of STS on the vascular network of the CAM was quantified utilizing the quantitative analysis of the fluorescent angiograms in ImageJ Macro (NIH, Bethesda, MD, USA) that was developed in our laboratory (28). Five eggs were dedicated to one condition with three images taken per one egg (altogether, 15 images per condition were analyzed).

### Thiosulfate measurement

Urine and plasma were collected and stored in –80°C until further analysis. Samples were kept on ice after thawing and centrifuged for 10 min at 15,000 rpm at 4°C. 10 μL supernatant were mixed with 15 μL buffer (160 mM HEPES, and 16 mM EDTA, pH 8.0), 15 μL 100% acetonitrile, and 3 μL 46 mM monobromobimane (mBBr) and incubated for 30 min in the dark at 20°C. Next 30 μL of 65 mM methanesulfonic acid was added as the stop solution and incubated again for 5 min in the dark at 20°C before centrifugation for 15 min at 15,000 rpm and 4°C. The supernatant was transferred to an HPLC vial and diluted 5-fold with running buffer A (0.25% acetic acid, pH 4.5). STS was analyzed by HPLC on a C18 reversed-phase column (EC 250/3 NUCLEODUR C18 HTec, 5 μm, MACHEREY-NAGEL) at 40°C and 0.9 mL/min in running buffer A. Against running buffer B (100% methanol) the following gradient profile was applied (Time [min]/Buffer B [%]): 0.00/15, 0.75/15, 2.65/23, 5.43/33, 6.03/37, 8.06/45, 8.44/65, 8.82/100, 11.34/100, 11.72/15, and 15.00/15%. STS was detected by fluorescence using an excitation at 380 nm and emission at 480 nm and quantified by peak area integration in comparison to standard.

### Hydrogen sulfide and persulfidation measurement

Free H_2_S was measured in cells using the SF_7_-AM fluorescent probe (29) (Sigma-Aldrich). The probe was dissolved in anhydrous DMF at 5 mM and used at 5 μM in serum-free EBM-2.

Global protein persulfidation was assessed on HUVEC grown on glass coverslips as previously described (23). Cells were incubated for 20 mins with 1 mM 4-Chloro-7-nitrobenzofurazan (NBF-Cl, Sigma) diluted in PBS. Then, cells were washed with PBS and fixed for 10 mins in ice-cold methanol. Coverslips were rehydrated in PBS, and incubated with 1 mM NBF-Cl for 1 h at 37°C. Cells were further incubated at 37°C for 1 h in Daz2-Cy5.5 solution prepared as previously described (23). Finally, coverslips were washed three times in methanol and two times in PBS, mounted in Vectashield mounting medium with DAPI, and visualized with a 90i Nikon fluorescence microscope.

### Wound healing assay

HUVEC were grown to confluence in a 12-well plate and a scratch wound was created using a sterile p200 pipette tip. Repopulation of the wound in presence of Mitomycin C was recorded by phase-contrast microscopy over 16 h in a Nikon Ti2-E live-cell microscope. The denuded area was measured at *t* = 0 h and *t* = 10 h after the wound using the ImageJ software. Data were expressed as a ratio of the healed area over the initial wound area.

### BrdU assay

HUVEC were grown at 80% confluence (5.10^3^ cells per well) on glass coverslips in a 24-well plate and starved overnight in serum-free medium (EBM-2, Lonza). Then, HUVEC were treated or not (ctrl) for 24 h in full medium (EGM-2, Lonza) in presence of 10 μM BrdU. All conditions were tested in parallel. Cells were fixed in ice-cold methanol 100 and immunostained for BrdU as previously described (23, 26, 30). Images were acquired using a Nikon Eclipse 90i microscope. BrdU-positive nuclei and total DAPI-positive nuclei were automatically detected using the ImageJ software (26).

### Seahorse

Glycolysis and Mitochondrial stress tests were performed on confluent HUVEC according to the manufacturer’s kits and protocols (Agilent Seahorse XF glycolysis stress test kit, Agilent Technologies, Inc.). 1 μM Oligomycin was used. Data were analyzed using the Seahorse Wave Desktop Software (Agilent Technologies, Inc., Seahorse Bioscience).

### Adenosine triphosphate assay

HUVEC were grown at 80% confluence (10.10^3^ cells per well) in a 12-well plate and starved overnight in serum-free medium (EBM-2, Lonza). Then, HUVEC were either treated or not (ctrl) with 3 mM STS for 24 h in full medium (EGM-2, Lonza), washed in ice-cold PBS and resuspended according to the ATP Assay Kit (Colorimetric/Fluorometric) (ab83355, Abcam).

### Immunohistochemistry

Ischemic and contralateral gastrocnemius muscle were collected and flash frozen in OCT 2 weeks post-op. OCT blocks were cut into 10 μM slides for immunostaining. Muscle sections were permeabilized in PBS supplemented with 2 wt.% BSA and 0.1 vol.% Triton X-100 for 30 min, blocked in PBS supplemented with 2 wt.% BSA and 0.1 vol.% Tween 20 for another 30 min, and incubated overnight using the antibodies described in Supplementary Table 1 diluted in the same buffer. The slides were then washed three times for 5 min in PBS supplemented with 0.1 vol.% Tween 20, and incubated for 1 h at room temperature with a mix of appropriate fluorescent-labeled secondary antibodies. Muscle fiber type staining were performed using the antibody developed by Sciaffino (31). EdU immunostaining was performed according to the manufacturer’s instructions (Click-iT™ Plus EdU Cell Proliferation Kit for Imaging, Alexa Fluor™ 594 dye, ThermoFischer). Images were acquired using a ZEISS Axioscan 7 Microscope Slide Scanner.

### Reverse transcription and quantitative polymerase chain reaction

HUVEC or grinded frozen gastrocnemius muscles were homogenized in Tripure Isolation Reagent (Roche, Switzerland), and total RNA was extracted according to the manufacturer’s instructions. After RNA Reverse transcription (Prime Script RT reagent, Takara), cDNA levels were measured by qPCR Fast SYBR™ Green Master Mix (Ref: 4385618, Applied Biosystems, ThermoFischer Scientific AG, Switzerland) in a Quant Studio 5 Real-Time PCR System (Applied Biosystems, ThermoFischer Scientific AG, Switzerland), using the primers given in the Supplementary Table 2.

### Statistical analyses

All experiments adhered to the ARRIVE guidelines and followed strict randomization. All experiments and data analysis were conducted in a blind manner using coded tags rather than actual group name. A power analysis was performed prior to the study to estimate sample-size. We hypothesized that STS would improve neovascularization by 20%. Using an SD at ±10% for the surgery and considering a power at 0.8, we calculated that *n* = 8 animals/group was necessary to validate a significant effect of the STS. Animals with pre-existing conditions (malocclusion, injury, and abnormal weight) were not operated or excluded from the experiments upon discovery during dissection. All experiments were analyzed using GraphPad Prism 9. Normal distribution of the data was assessed using Kolmogorov-Smirnov tests. All data had a normal distribution. Unpaired bilateral Student’s *t*-tests, or one or two-ways ANOVA were performed followed by multiple comparisons using *post hoc t*-tests with the appropriate correction for multiple comparisons.

## Results

### Sodium thiosulfate promotes reperfusion and muscle recovery in a mouse model of hindlimb ischemia

**FIGURE 1:**
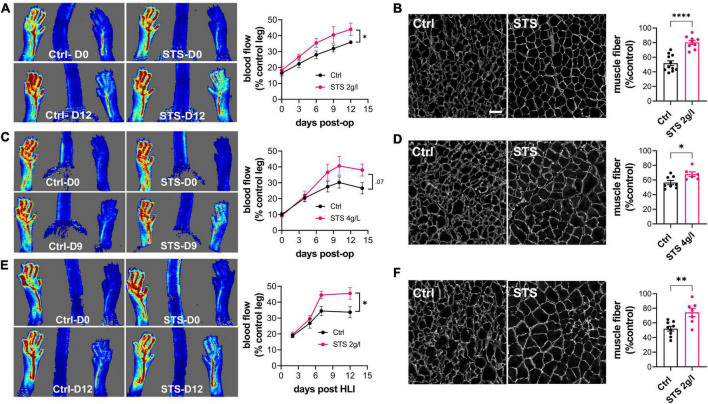
Sodium thiosulfate (STS) promotes revascularization and muscle recovery in a mouse model of hindlimb ischemia. Doppler imaging **(A,C,E)** and laminin immunostaining in the gastrocnemius muscle **(B,D,F)** of WT male mice submitted to HLI and treated or not (Ctrl) with 2 g/L STS **(A,B)** or 4 g/L STS **(C,D)**, or LDLR^– /–^ mice treated with 2 g/L STS **(E,F)**. **(A,C,E)** Data are mean ± SEM of 8–12 animals per group. *p < 0.05 as determined by repeated measures Mixed-effects model (REML). **(B,D,F)** Data are mean ± SEM of 6–12 animals per group. *p < 0.05, **p < 0.01; ****p < 0.0001 as determined by bilateral unpaired *t*-test. Scale bar 100 μm.

To test the benefits of STS on vascular recovery, hindlimb ischemia (HLI) was induced by transection of the femoral artery, which leads to ischemia-induced muscle damage. Laser Doppler imaging showed that HLI reduced blood flow by >80% both in Ctrl and mice treated with 2 or 4 g/L of STS. Doppler imaging further revealed that 2 g/L STS improved reperfusion (Figure 1A), while 4 g/L tended to increase blood flow compared to Ctrl mice (Figure 1C). Morphological analysis of the gastrocnemius muscle 14 days post-surgery further showed that STS reduced muscle damaged as assessed by the mean muscle fiber cross-sectional area (Figures 1B,D). We also performed the HLI on hypercholesterolemic LDLR^–/–^ mice fed for 3 weeks with a cholesterol-rich diet, a model closer to the dyslipidaemia state of PAD patients. 2 g/L STS also increased reperfusion (Figure 1E) and reduced muscle damage (Figure 1F) in this diseased model.

### Sodium thiosulfate leads to enzymatic production of hydrogen sulfide and increases protein persulfidation

**FIGURE 2:**
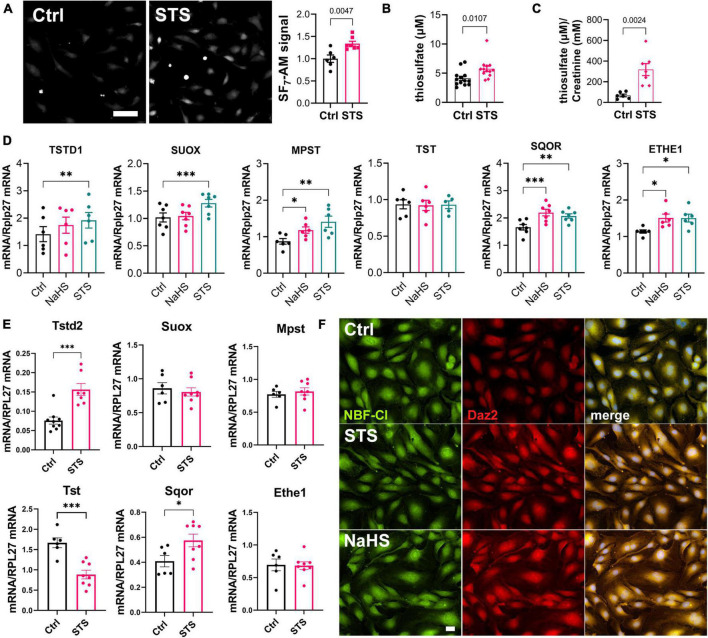
Sodium thiosulfate (STS) leads to enzymatic production of hydrogen sulfide (H_2_S) and increases protein persulfidation. **(A)** H_2_S release measured by the SF_7_-AM probe in HUVEC exposed for 90 min to STS. Data are mean ± SEM of six independent experiments. *p < 0.05 as determined by bilateral unpaired *t*-test. Scale bar 20 μm. **(B)** Plasma levels of thiosulfate in mice treated or not (Ctrl) for 2 weeks with 4 g/L STS. Data are mean ± SEM of 12 animals per group. *p* = 0.011 as determined by bilateral- unpaired *t*-test. **(C)** Urine levels of thiosulfate, normalized to creatinine levels, in mice treated or not (Ctrl) for 2 weeks with 4 g/L STS. Data are mean ± SEM of 6 or 7 animals per group. *p* = 0.0035 as determined by bilateral- unpaired *t*-test. **(D)** mRNA expression in HUVEC exposed for 4 h to NaHS (100 μM) or STS (3 mM). Data are mean ± SEM of six independent experiments. *p < 0.05, **p < 0.01; ***p < 0.001 as determined by repeated measures one-way ANOVA with Dunnett’s *post hoc* test. **(E)** mRNA expression in gastrocnemius muscle of mice treated for 1 week with 4 g/L STS. Data are mean ± SEM of 6–8 animals per group. *p < 0.05, **p < 0.01; ***p < 0.001 as determined by bilateral- unpaired *t*-test. **(F)** *In situ* labeling of intracellular protein persulfidation assessed by DAz-2:Biotin-Streptavidin-584 (red), normalized to NBF-adducts fluorescence (green), in HUVEC exposed for 4 h to NaHS (100 μM) or STS (3 mM).

We previously demonstrated that STS behaves as a H_2_S donor (23, 32). To test whether STS releases detectable amounts of H_2_S in HUVECs, we used the H_2_S specific probe SF_7_-AM (29). SF_7_-AM signal was monitored in HUVECs 90 min post addition of 15 mM STS, and showed a 50% increase in SF_7_-AM signal (Figure 2A). *In vivo*, STS treatment via the water bottle at 4 g/L significantly increased circulating levels of thiosulfate (Figure 2B) and urinary excretion of mM amounts of thiosulfate (Figure 2C). Thiosulfate is an intermediate of sulfur metabolism metabolized by the H_2_S biosynthetic pathway and sulfide-oxidizing unit (33, 34). A 4 h treatment with STS, but not NaHS, increased the mRNA expression of sulfite oxidase (SUOX), thiosulfate sulfurtransferase-like domain containing 1 (TSTD1), mercaptopyruvate sulfurtransferase (MPST), but not thiosulfate sulfurtransferase (TST) in HUVECs (Figure 2D). However, neither STS nor NaHS influenced the mRNA expression of H_2_S-generating enzymes CBS and CSE (Supplementary Figure 1). Of note, both NaHS and STS increased the expression of the mitochondrial H_2_S-detoxifing enzymes sulfide quinone oxidoreductase (SQOR) and persulfide dioxygenase ETHE1 (Figure 2D). In gastrocnemius muscles from mice treated with 4 g STS for 1 week, STS treatment increased mRNA expression of Tstd2 and Sqor, whereas the expression of Tst was decreased. STS did not affect the mRNA expression of Suox, Mpst, and Ethe1 (Figure 2E). H_2_S signals through post-translational modifications of reactive cysteine residues by persulfidation (35, 36). Both STS and NaHS increased protein persulfidation as measured by DAZ-2-Cy5.5 labeling of persulfide residues in HUVEC treated for 4 h with 100 μM NaHS or 15 mM STS (Figure 2F).

### Sodium thiosulfate promotes arteriogenesis and angiogenesis *in vivo*

**FIGURE 3:**
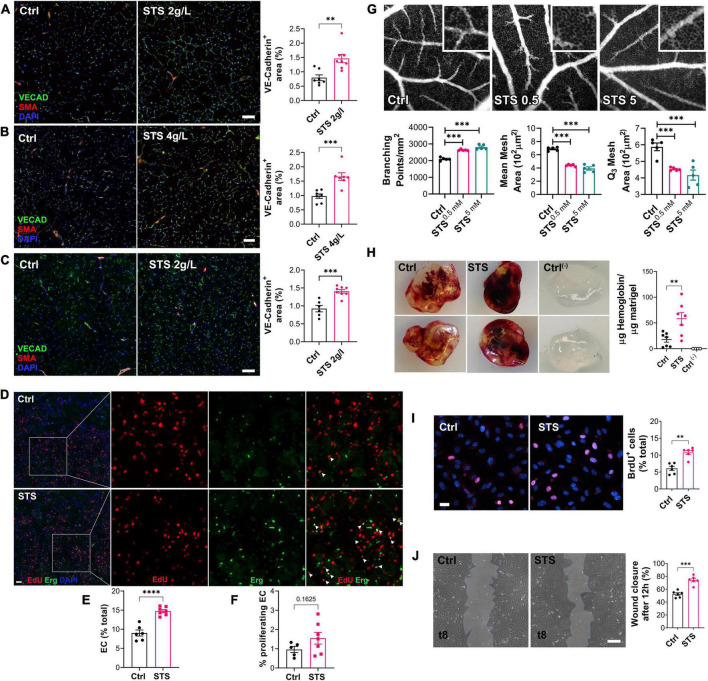
Sodium thiosulfate (STS) promotes angiogenesis *in vivo* in several models. **(A–C)** VE-cadherin (VECAD; green), smooth muscle actin (SMA; red) and nuclei (DAPI; blue) immunostaining in gastrocnemius muscle from WT male mice submitted to HLI and treated or not (Ctrl) with 2 g/L STS **(A)** or 4 g/L STS **(B)**, or LDLR^– /–^ mice treated with 2 g/L STS **(C)**. Representative images and quantification of the VECAD staining in 6–9 animals per group. Data are mean ± SEM. **p < 0.01, ***p < 0.001 as determined by bilateral unpaired *t*-test. **(D)** EdU (red), ERG (green) and nuclei (blue) immunostaining in ischemic muscle of WT mice treated with 2 g/L STS 4 days after HLI. Scale bar represent 100 μm. Insets are 3-fold magnification of left images. **(E)** Percentage of endothelial cells (ERG+/total nuclei count). **(F)** Percentage of proliferating endothelial cells (EdU+/ERG+). Data are mean ± SEM. *p < 0.05, ****p < 0.0001 as determined by bilateral unpaired *t*-test. **(G)** Representative fluorescein-dextran fluorescence angiographies at EDD13, 48 h after topical treatment with 0.9% NaCl (Ctrl), or STS (0.5 or 5 mM final). Representative images and quantification of the vascular network from 5 eggs per group. Data are mean ± SEM. ***p < 0.001 as determined by one-way ANOVA with Tukey’s *post hoc* test. **(H)** Matrigel plugs supplemented or not (ctrl-) with VEGF_135_ ± STS (15 mM) 1 week post implantation. Representative plugs and hemoglobin content normalized to plug weight. Quantification of 4–8 animals per group. Data are mean ± SEM. **p < 0.01 as determined by one-way ANOVA with Tukey’s *post hoc* test. **(I)** HUVEC proliferation assessed by BrdU incorporation and expressed as BrdU positive cells (pink) over DAPI positive nuclei. Data shown as mean ± SEM of six independent experiments. **p < 0.01 as determined by bilateral unpaired *t*-test. **(J)** HUVEC migration was assessed by wound healing assay in presence of Mitomycin C and expressed as the percentage of wound closure after 10 h. Data shown as mean ± SEM of six independent experiments. **p < 0.01, ***p < 0.001 as determined by bilateral unpaired *t*-test.

To test whether STS increased blood perfusion via improved micro-vessel regeneration, we determined the micro-vessel density in the gastrocnemius muscle using VE-Cadherin immunofluorescent staining of WT mice. Both 2 and 4 g/L STS treatment increased the micro-vessel density as compared to Ctrl mice 14 days after HLI (Figures 3A,B). STS treatment also increased the micro-vessel density in LDLR^–/–^ mice (Figure 3C). EdU/Erg immunofluorescent staining on ischemic muscles 4 days after ischemia showed that STS increased the percentage of EC (Figures 3D,E) and proliferating EC in WT mice (Figures 3D,F). Then, we assessed the effect of STS on angiogenesis *in ovo* using the Chicken chorioallantoic membrane (CAM) assay and the Matrigel plug assay. STS was applied topically to achieve 0.5 or 5 mM at embryonic development day 11 (EDD11). Observations at EDD13 revealed that STS promoted the capillary formation measured as the relative number of branching points/mm^2^, relative mean mesh size and Q3 mesh area of the vessel network (Figure 3G). The addition of 15 mM STS in Matrigel plugs also promoted VEGF-induced angiogenesis as assessed by hemoglobin content 7 days after subcutaneous injection in the mouse (Figure 3H). To investigate the effect of STS on EC directly, we assessed the proliferation and migration of primary human umbilical vein endothelial cells (HUVECs). STS increased HUVEC proliferation (Figure 3I), similarly to the H_2_S donor salt NaHS and the slow-releasing H_2_S donor GYY4137 (Supplementary Figure 2). STS also promoted HUVEC migration in a wound healing assay (Figure 3J). To further study endothelial function, we investigated known markers of endothelial function (37). A 4 h treatment with 3 mM STS increased eNOS and VEGFR2 mRNA expression in HUVEC (Supplementary Figure 3A). Western blot analysis from mice who underwent hindlimb ischemia treated with STS for 2 weeks revealed that 2 g/L STS increased the ratio of P-eNOS over eNOS and increased the ratio of P-VEGFR2/VEGFR2 levels in the ischemic gastrocnemius muscle (Supplementary Figure 3B). Of note, this chronic long-term treatment decreased total eNOS and VEGFR2 protein levels.

### Sodium thiosulfate limits inflammation and muscle damage 4 days after ischemia

**FIGURE 4:**
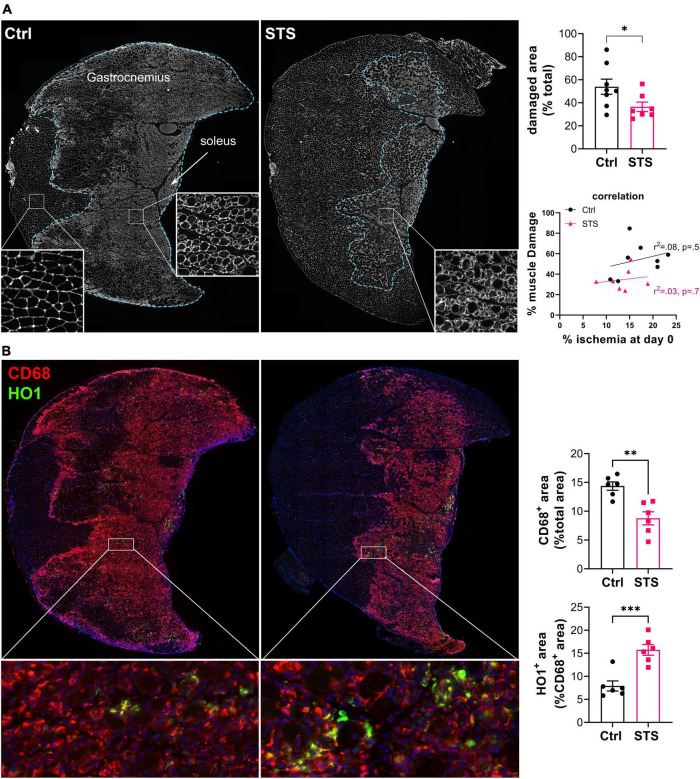
Sodium thiosulfate (STS) limits inflammation and muscle damage 4 days after ischemia. **(A)** *Left panel*: Representative images of gastrocnemius and soleus muscle of WT mice treated or not (Ctrl) with 4 g/L STS, stained for laminin (white). Damaged area delimited by the blue dotted line. *Right upper panel*: Quantification of the damaged area, expressed as a percentage of the total muscle area. Data are mean ± SEM. *p < 0.05 as determined by unpaired *t*-test. *Right lower panel*: correlation between muscle damage and ischemia after the surgery. Data analyzed by Pearson correlation. **(B)** *Left panel:* Representative images of gastrocnemius and soleus muscle of WT mice treated or not (Ctrl) with 2 g/L STS, stained for CD68 (red) and HO1 (green). *Right panel*: CD68 and HO1 positive area quantification. Data are mean ± SEM. **p < 0.01 ***p < 0.001 as determined by bilateral unpaired *t*-test.

After ischemia, inflammation plays a major role in muscle function and repair. Specifically, macrophages shifted toward the M2 phenotype are instrumental in arteriogenesis following ischemia (38). STS limited muscle damage in the gastrocnemius muscle of mice 4 days after HLI, as assessed by laminin staining (Figure 4A). Of note, there is no correlation between muscle damage and percentage of ischemia at day 0 (Figure 4A). Decreased muscle damage was accompanied by a significant reduction in macrophage infiltration, as assessed by CD68+ staining. Furthermore, STS increased the percentage of HO1^+^ CD68^+^ macrophages, a marker of M2 pro-resolving macrophages (Figure 4B). Given that STS improves muscle repair, we further studied muscle fiber type distribution pre and 14 days post-op in mice treated or not with STS (Supplementary Figure 4). Pre-op immunostaining of type I, type IIA and type IIB fibers showed that the soleus was composed of 60% type I slow (I, Myh7 in blue) and 40% of fast (IIA, Myh2 in red) oxidative fibers. The gastrocnemius was made of more than 80% of fast glycolytic IIB fibers (type IIB, Myh4 in green), and 10% of type IIA located toward the soleus. The remaining were mainly negative fibers. Type I fibers constituted less than 1% of the total fibers in the gastrocnemius. Post-ischemia in the soleus, which suffered the most from ischemic injury, hybrid type I/IIA fibers (red blue = purple) almost completely replaced type I fibers. In the gastrocnemius, IIB fibers were significantly reduced, while type IIA increased to 15% and hybrid type I/IIA fibers appear. STS increased the proportion of hybrid type I/IIA fibers in both muscles (Supplementary Figure 4).

### Sodium thiosulfate inhibits mitochondrial respiration and increases glycolysis and adenosine triphosphate production in HUVECs

**FIGURE 5:**
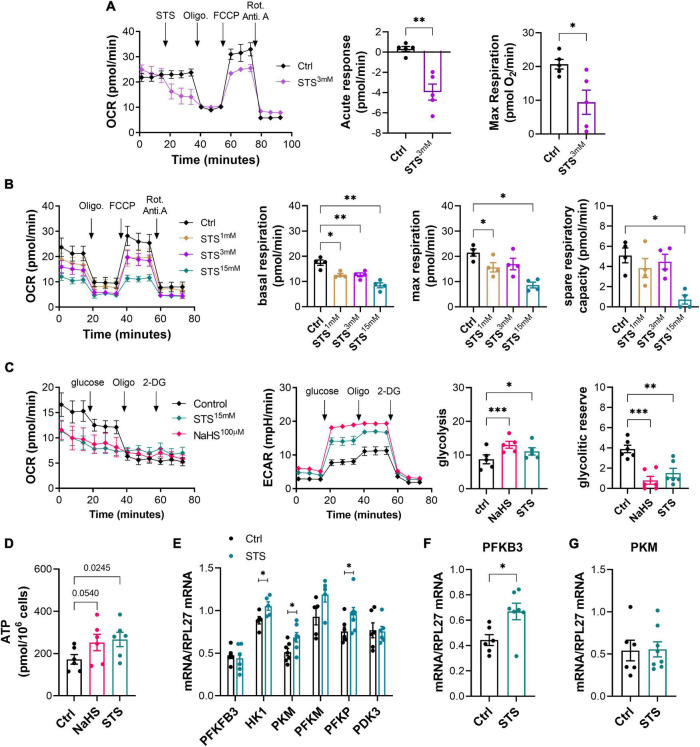
Sodium thiosulfate (STS) inhibits mitochondrial respiration and increases glycolysis and adenosine triphosphate (ATP) production in HUVECs. **(A)** Acute effect of STS on respiration in a mitochondrial stress test assay using HUVEC. *Left panel:* representative traces of oxygen consumption rate (OCR). *Right panels:* quantitative assessment of decreased OCR in response to STS injection and maximal respiration upon FCCP injection. Data are mean ± SEM of five independent experiments. *p < 0.05, **p < 0.01 as determined by bilateral unpaired *t*-test. **(B)** Mitochondrial stress test in HUVEC pre-treated for 4 h with increasing concentration of STS, as indicated. Data are mean ± SEM of 54 independent experiments. *p < 0.05, **p < 0.01 as determined by repeated measures mixed-effects model (REML) followed by Dunnett’s multiple comparisons tests. **(C)** Glycolysis stress test in HUVECs pre-treated for 4 h with 3 mM STS or 100 μM NaHS. Glycolysis is measured by extracellular acidification rate (ECAR). Data expressed as mean ± SEM of five independent experiments. *p < 0.05, **p < 0.01, ***p < 0.001 as determined by repeated measures mixed-effects model (REML) followed by Dunnett’s multiple comparisons tests. **(D)** Adenosine triphosphate (ATP) production in HUVEC treated for 24 h with 100 μM NaHS or 3 mM STS. Statistics are *p*-values determined by bilateral unpaired *t*-test. **(E)** Normalized mRNA levels of key glycolysis genes in HUVEC pre-treated for 4 h with 15 mM STS. Data are mean ± SEM of seven independent experiments. *p < 0.05 as determined by bilateral unpaired *t*-test. **(F,G)** PFKFB3 **(F)** and PKM **(G)** mRNA levels in the gastrocnemius muscles of mice treated or not with 4 g/L STS for 1 week. Data are mean ± SEM of seven animals per group. *p < 0.05 as determined by bilateral unpaired *t*-test.

ECs are glycolytic, thus favoring glycolysis for ATP production. This key feature allows EC to proliferate and migrate in hypoxic conditions in the context of angiogenesis (39, 40). H_2_S blocks mitochondrial respiration through inhibition of the complex IV of the mitochondria, which increases compensatory glycolysis in EC and promotes angiogenesis (17). To test the effect of STS on mitochondrial respiration, we performed a mitochondrial stress test in a seahorse apparatus. 3 mM STS rapidly reduced oxygen consumption rate (OCR) in HUVEC (Figure 5A). A 4-h pre-treatment with STS dose-dependently inhibited mitochondrial respiration in HUVEC, leading to reduced basal and max respiration and ATP production in that assay (Figure 5B). To measure the cell’s glycolytic reserve, i.e., the ability to increase glycolysis upon inhibition of respiration, we then performed a glycolysis stress test on HUVEC pre-treated for 4 h with STS or NaHS. Inhibition of mitochondrial respiration using oligomycin promoted glycolysis (ECAR) in HUVEC (Figure 5C). The donors increased basal glycolysis in HUVEC, thereby reducing the glycolytic reserve (Figure 5C). We further confirmed that an 8-h treatment with 3 mM STS increased ATP production in HUVECs (Figure 5D). In EC, the enzyme PFKFB3 tightly regulates glycolysis (39). A 4-h pre-treatment with 15 mM STS stimulated mRNA expression of key glycolysis genes in HUVEC (Figure 5E). In the gastrocnemius, 1 week treatment with 4 g/L of STS significantly increased the mRNA expression of PFKFB3 (Figure 5F), whereas the mRNA of PKM was not affected (Figure 5G).

### Sodium thiosulfate-induced HUVEC proliferation requires glycolysis

**FIGURE 6:**
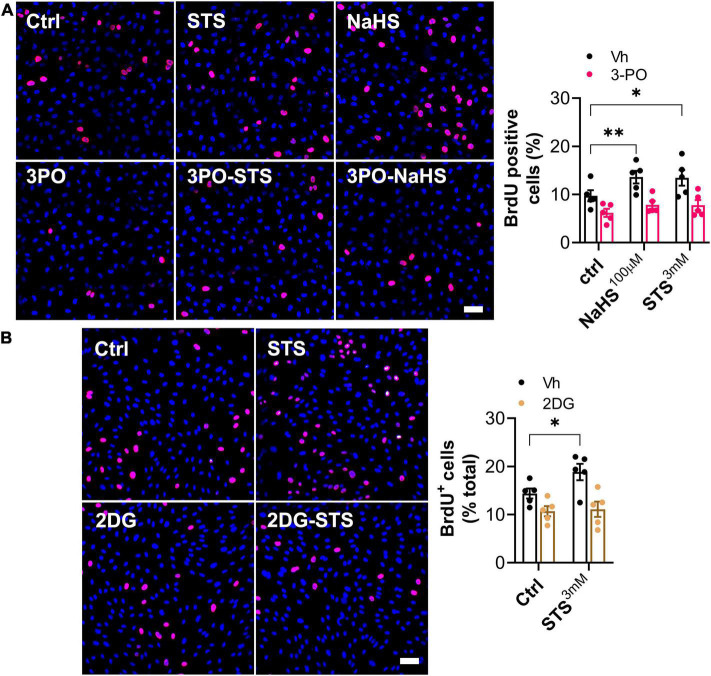
Sodium thiosulfate (STS)-induced HUVEC proliferation requires glycolysis. **(A,B)** HUVEC proliferation (BrdU incorporation) in cells treated for 8 h with 100 μM NaHS, 3 mM STS ± 15 μM PFKFB3 inhibitor (3-PO) or 6 mM 2 deoxy-glucose (2DG), or their respective vehicle (Vh). *Left panels:* representative images. *Right panels:* Data are mean ± SEM of ratio of BrdU positive cells (pink) over DAPI positive nuclei (blue) in five independent experiments. *p < 0.05 as determined by repeated measures two-way ANOVA with Dunnett’s *post hoc* test.

To confirm that the effect of STS on angiogenesis is glycolysis-dependent, we assessed the proliferation of HUVECs, in presence or not of the glycolysis inhibitor 3PO or the glucose competitor 2-deoxy-glucose (2-DG). Both 3PO (Figure 6A) and 2-DG (Figure 6B) treatment reduced basal HUVEC proliferation and fully abolished the positive effect of STS on proliferation.

## Discussion

Despite potent cardiovascular benefits, there is no clinically approved H_2_S-releasing molecule. STS does not directly release H_2_S, but provide thiosulfate, which can further lead to formation of H_2_S and polysulfides via rhodanese activity and the reverse transsulfuration pathway (20). Our study demonstrate that STS treatment promotes the enzymatic metabolism of thiosulfate through both the H_2_S biosynthetic pathway and sulfide oxidizing unit (33, 34), yielding minutes amounts of H_2_S with measurable effects on global protein persulfidation. The fact that STS increased protein persulfidation suggests that STS works similarly as a H_2_S donor (41). This is in line with previous studies showing that thiosulfate can be metabolized to H_2_S through sulfite formation or bound sulfane sulfur release (20, 32, 42–44).

In line with previous studies using other sources of H_2_S (8–10), STS increases revascularization *in vivo* following hindlimb ischemia. Reminiscent of other studies performed with NaHS (13, 45, 46), STS also promotes angiogenesis in the CAM model and specifically promotes VEGF-driven sprouting angiogenesis in matrigel plug implants. At the cellular levels, STS stimulates EC proliferation and migration *in vitro* in cultured endothelial cells, and *in vivo* in the ischemic muscle, leading to increased capillary density and blow flow.

The fact that STS promoted VEGF-dependent sprouting angiogenesis in matrigel plugs, but also neovascularization in the HLI model, suggest that STS/H_2_S may act on different levels to promote neovascularization. First, H_2_S is known to promote angiogenesis via stimulation of the VEGFR2 and NO pathway (16, 47–49). In this study, we also observed that STS stimulates the VEGFR2 and eNOS pathways *in vitro* and *in vivo* in the muscle post HLI. Second, we previously showed that H_2_S promotes the metabolic switch in EC to favor glycolysis, and this mechanism promotes VEGF-induced EC migration (17). Indeed, EC rely mostly on glycolysis for energy production and further upregulate glycolysis to fuel migration and proliferation during angiogenesis (39, 40). Here, STS inhibited mitochondrial respiration, inducing a compensatory increase in glycolysis, which seems instrumental to observe STS-induced EC proliferation. *In vivo* data also suggest that STS treatment modulates the expression of genes involved in glycolysis. Third and last, neovascularization in the HLI model is mediated by both arteriogenesis and angiogenesis. Arteriogenesis is driven by shear stress and requires macrophages to achieve proper vessel remodeling (50, 51). In particular, pro-resolving macrophages shifted toward the M2 phenotype are instrumental for arteriogenesis following skeletal ischemia (52). In line with a role of macrophages in early recovery and arteriogenesis, we observed massive macrophage infiltration in the muscle 4 days after HLI. Our data indicate an anti-inflammatory effect of STS, and suggest a shift toward HO-1+ pro-repair M2 macrophages (53). Interestingly, H_2_S possesses anti-inflammatory properties (54) and was suggested to promote the shift toward the M2 phenotype in the context of atherosclerosis (55). Given that the increase in HO1+ macrophages is accompanied by a decrease in total macrophages, the exact impact of STS on macrophage polarization remains to be clarify to determine whether STS inhibits inflammation in general, or specifically promotes the M2 phenotype. Reduced inflammation could a bystander effect of accelerated neovascularization. Further studies remain to be performed to test the respective role of metabolic reprogramming, VEGF potentiation and anti-inflammatory properties of STS to improve recovery in the HLI model. Overall, we propose that STS acts at several levels to promote both arteriogenesis and angiogenesis.

To replicate some of the comorbidities of PAD patients, we further confirmed that STS improved neovascularization in hypercholesterolemic mice. However, the HLI model remains a model of acute ischemia, with limited impairment in limb function and fast reperfusion to asymptomatic levels, even in hypercholesterolemic mice. In addition, further studies are required to assess the function and leakiness of neovessels in response to STS.

Sodium thiosulfate is clinically approved and safe in gram quantities in humans. We recently showed that oral STS at 4 g/L has no toxicity on mice (23). Here, we show that STS at 4 g/L leads to a modest increase in circulating thiosulfate levels, while most of the thiosulfate absorbed in probably eliminated in the urine. Additional experiments are required to assess STS accumulation and distribution in tissues. The fact that STS was more potent in stimulating revascularization at 2 g/L than at 4 g/L suggest a narrow therapeutic range for the pro-angiogenic effects of STS.

In conclusion, STS, a molecule with high translational potential since already approved clinically, promotes EC proliferation and recovery after hindlimb ischemia, both in WT and hypercholesterolemic LDLR^–/–^ mice. STS promotes EC proliferation in a glycolysis-dependent manner. We recently showed that STS inhibits intimal hyperplasia, which is the bane of all surgical revascularization (23). These findings suggest that STS holds strong potential to promote vascular repair in PAD patients, while limiting intimal hyperplasia. Altogether, this calls for further pre-clinical studies in the large animal, and prospective clinical trials in patients.

## Data availability statement

The raw data supporting the conclusions of this article will be made available by the authors, without undue reservation.

## Ethics statement

This animal study was reviewed and approved by Cantonal Veterinary Office (SCAV-EXPANIM; authorization number: 3504).

## Author contributions

FA, AL, and SD designed the study. FA, JJ, QG, DM, C-YF, and ML performed the experiments. FA, JJ, DM, ML, GS, GW, and SD analyzed the data. FA, DM, AL, and SD wrote the manuscript. FA and DM finalized the manuscript. All authors critically revised the manuscript and approved the submitted version.

## References

[1] Eraso LH Fukaya E Mohler ER III Xie D Sha D Berger JS. Peripheral arterial disease, prevalence and cumulative risk factor profile analysis. *Eur J Prevent Cardiol.* (2014) 21:704–11. 10.1177/2047487312452968 22739687 PMC4436703

[2] Song P Rudan D Zhu Y Fowkes FJI Rahimi K Fowkes FGR Global, regional, and national prevalence and risk factors for peripheral artery disease in 2015: an updated systematic review and analysis. *Lancet Glob Health.* (2019) 7:e1020–30. 10.1016/S2214-109X(19)30255-4 31303293

[3] Arao K Yasu T Endo Y Funazaki T Ota Y Shimada K Investigators of the A-A, lipid lowering with pitavastatin evaluation study in N. effects of pitavastatin on walking capacity and CD34(+)/133(+) cell number in patients with peripheral artery disease. *Heart Vessels.* (2017) 32:1186–94. 10.1007/s00380-017-0988-1 28466409 PMC5614906

[4] Shahin Y Barnes R Barakat H Chetter IC. Meta-analysis of angiotensin converting enzyme inhibitors effect on walking ability and ankle brachial pressure index in patients with intermittent claudication. *Atherosclerosis.* (2013) 231:283–90. 10.1016/j.atherosclerosis.2013.09.037 24267241

[5] Lane R Ellis B Watson L Leng GC. Exercise for intermittent claudication. *Cochrane Database Syst Rev.* (2014) 7:CD000990. 10.1002/14651858.CD000990.pub3 25037027

[6] Iyer SR Annex BH. Therapeutic angiogenesis for peripheral artery disease: lessons learned in translational science. *JACC Basic Transl Sci.* (2017) 2:503–12. 10.1016/j.jacbts.2017.07.012 29430558 PMC5802410

[7] Cirino G Szabo C Papapetropoulos A. Physiological roles of hydrogen sulfide in mammalian cells, tissues and organs. *Physiol Rev.* (2022) 28:2021. 10.1152/physrev.00028.2021 35435014

[8] Majumder A Singh M George AK Behera J Tyagi N Tyagi SC. Hydrogen sulfide improves postischemic neoangiogenesis in the hind limb of cystathionine-beta-synthase mutant mice via PPAR-gamma/VEGF axis. *Physiol Rep.* (2018) 6:e13858. 10.14814/phy2.13858 30175474 PMC6119702

[9] Wang MJ Cai WJ Li N Ding YJ Chen Y Zhu YC. The hydrogen sulfide donor NaHS promotes angiogenesis in a rat model of hind limb ischemia. *Antioxid Redox Signal.* (2010) 12:1065–77. 10.1089/ars.2009.2945 19842913

[10] Rushing AM Donnarumma E Polhemus DJ Au KR Victoria SE Schumacher JD Effects of a novel hydrogen sulfide prodrug in a porcine model of acute limb ischemia. *J Vasc Surg.* (2019) 69:1924–35. 10.1016/j.jvs.2018.08.172 30777693 PMC6548642

[11] Annex BH Cooke JP. New directions in therapeutic angiogenesis and arteriogenesis in peripheral arterial disease. *Circ Res.* (2021) 128:1944–57. 10.1161/CIRCRESAHA.121.318266 34110899 PMC8538391

[12] Yuan S Kevil CG. Nitric oxide and hydrogen sulfide regulation of ischemic vascular remodeling. *Microcirculation.* (2016) 23:134–45. 10.1111/micc.12248 26381654 PMC6939643

[13] Papapetropoulos A Pyriochou A Altaany Z Yang G Marazioti A Zhou Z Hydrogen sulfide is an endogenous stimulator of angiogenesis. *Proc Natl Acad Sci USA.* (2009) 106:21972–7. 10.1073/pnas.0908047106 19955410 PMC2799889

[14] Polhemus DJ Kondo K Bhushan S Bir SC Kevil CG Murohara T Hydrogen sulfide attenuates cardiac dysfunction after heart failure via induction of angiogenesis. *Circ Heart Fail.* (2013) 6:1077–86. 10.1161/CIRCHEARTFAILURE.113.000299 23811964 PMC3819451

[15] Jang H Oh MY Kim YJ Choi IY Yang HS Ryu WS Hydrogen sulfide treatment induces angiogenesis after cerebral ischemia. *J Neurosci Res.* (2014) 92:1520–8. 10.1002/jnr.23427 24939171

[16] Tao BB Liu SY Zhang CC Fu W Cai WJ Wang Y VEGFR2 functions as an H2S-targeting receptor protein kinase with its novel Cys1045-Cys1024 disulfide bond serving as a specific molecular switch for hydrogen sulfide actions in vascular endothelial cells. *Antioxid Redox Signal.* (2013) 19:448–64. 10.1089/ars.2012.4565 23199280 PMC3704125

[17] Longchamp A Mirabella T Arduini A MacArthur MR Das A Trevino-Villarreal JH Amino acid restriction triggers angiogenesis via GCN2/ATF4 regulation of VEGF and H2S production. *Cell.* (2018) 173:117–29.e14. 10.1016/j.cell.2018.03.001 29570992 PMC5901681

[18] Bebarta VS Brittain M Chan A Garrett N Yoon D Burney T Sodium nitrite and sodium thiosulfate are effective against acute cyanide poisoning when administered by intramuscular injection. *Ann Emerg Med.* (2017) 69:718–25.e4. 10.1016/j.annemergmed.2016.09.034 28041825 PMC5446299

[19] Nigwekar SU Thadhani R Brandenburg VM. Calciphylaxis. *N Engl J Med.* (2018) 378:1704–14. 10.1056/NEJMra1505292 29719190

[20] Olson KR Deleon ER Gao Y Hurley K Sadauskas V Batz C Thiosulfate: a readily accessible source of hydrogen sulfide in oxygen sensing. *Am J Physiol Regul Integr Comp Physiol.* (2013) 305:R592–603. 10.1152/ajpregu.00421.2012 23804280

[21] Snijder PM Frenay AR de Boer RA Pasch A Hillebrands JL Leuvenink HG Exogenous administration of thiosulfate, a donor of hydrogen sulfide, attenuates angiotensin II-induced hypertensive heart disease in rats. *Br J Pharmacol.* (2015) 172:1494–504. 10.1111/bph.12825 24962324 PMC4369259

[22] Ravindran S Kurian GA. Effect of sodium thiosulfate postconditioning on ischemia-reperfusion injury induced mitochondrial dysfunction in rat heart. *J Cardiovasc Transl Res.* (2018) 11:246–58. 10.1007/s12265-018-9808-y 29721767

[23] Macabrey D Longchamp A MacArthur MR Lambelet M Urfer S Deglise S Sodium thiosulfate acts as a hydrogen sulfide mimetic to prevent intimal hyperplasia via inhibition of tubulin polymerisation. *EBioMed.* (2022) 78:103954. 10.1016/j.ebiom.2022.103954 35334307 PMC8941337

[24] Ishibashi S Brown MS Goldstein JL Gerard RD Hammer RE Herz J. Hypercholesterolemia in low density lipoprotein receptor knockout mice and its reversal by adenovirus-mediated gene delivery. *J Clin Invest.* (1993) 92:883–93. 10.1172/JCI116663 8349823 PMC294927

[25] National Research Council. *Committee for the Update of the Guide for the Care and Use of Laboratory Animals., Institute for Laboratory Animal Research.* Washington, D.C: National Academies Press (2011).

[26] Longchamp A Kaur K Macabrey D Dubuis C Corpataux JM Deglise S Hydrogen sulfide-releasing peptide hydrogel limits the development of intimal hyperplasia in human vein segments. *Acta Bio.* (2019) 97:374–84. 10.1016/j.actbio.2019.07.042 31352106 PMC6801028

[27] Joniova J Wagnieres G. Catechin reduces phototoxic effects induced by protoporphyrin IX-based photodynamic therapy in the chick embryo chorioallantoic membrane. *J Biomed Opt.* (2020) 25:1–9. 10.1117/1.JBO.25.6.063807 PMC7013152 32052612

[28] Nowak-Sliwinska P Ballini JP Wagnieres G van den Bergh H. Processing of fluorescence angiograms for the quantification of vascular effects induced by anti-angiogenic agents in the CAM model. *Microvasc Res.* (2010) 79:21–8. 10.1016/j.mvr.2009.10.004 19857502

[29] Lin VS Lippert AR Chang CJ. Cell-trappable fluorescent probes for endogenous hydrogen sulfide signaling and imaging H2O2-dependent H2S production. *Proc Natl Acad Sci USA.* (2013) 110:7131–5. 10.1073/pnas.1302193110 23589874 PMC3645565

[30] Macabrey D Deslarzes-Dubuis C Longchamp A Lambelet M Ozaki CK Corpataux JM Hydrogen sulphide release via the angiotensin converting enzyme inhibitor zofenopril prevents intimal hyperplasia in human vein segments and in a mouse model of carotid artery stenosis. *Eur J Vasc Endovasc Surg.* (2022) 63:336–46. 10.1016/j.ejvs.2021.09.032 34916111

[31] Schiaffino S Gorza L Sartore S Saggin L Ausoni S Vianello M Three myosin heavy chain isoforms in type 2 skeletal muscle fibres. *J Muscle Res Cell Motil.* (1989) 10:197–205. 10.1007/BF01739810 2547831

[32] Macabrey D Longchamp A MacArthur MR Lambelet M Urfer S Corpataux J-M Sodium thiosulfate acts as an H_2_S mimetic to prevent intimal hyperplasia via inhibition of tubulin polymerization. *bioRxiv* [preprint] (2021). 10.1101/2021.09.09.459573 PMC8941337 35334307

[33] Paul BD Snyder SH Kashfi K. Effects of hydrogen sulfide on mitochondrial function and cellular bioenergetics. *Redox Biol.* (2021) 38:101772. 10.1016/j.redox.2020.101772 33137711 PMC7606857

[34] Olson KR. H2S and polysulfide metabolism: conventional and unconventional pathways. *Biochem Pharmacol.* (2018) 149:77–90. 10.1016/j.bcp.2017.12.010 29248597

[35] Li Z Polhemus DJ Lefer DJ. Evolution of hydrogen sulfide therapeutics to treat cardiovascular disease. *Circ Res.* (2018) 123:590–600. 10.1161/CIRCRESAHA.118.311134 30355137

[36] Zivanovic J Kouroussis E Kohl JB Adhikari B Bursac B Schott-Roux S Selective persulfide detection reveals evolutionarily conserved antiaging effects of s-sulfhydration. *Cell Metab.* (2019) 30:1152–70.e13. 10.1016/j.cmet.2019.10.007 31735592 PMC7185476

[37] Heiss C Rodriguez-Mateos A Kelm M. Central role of eNOS in the maintenance of endothelial homeostasis. *Antioxid Redox Signal.* (2015) 22:1230–42. 10.1089/ars.2014.6158 25330054 PMC4410282

[38] De Bock K Georgiadou M Schoors S Kuchnio A Wong BW Cantelmo AR Role of PFKFB3-driven glycolysis in vessel sprouting. *Cell.* (2013) 154:651–63. 10.1016/j.cell.2013.06.037 23911327

[39] Zecchin A Kalucka J Dubois C Carmeliet P. How endothelial cells adapt their metabolism to form vessels in tumors. *Front Immunol.* (2017) 8:1750. 10.3389/fimmu.2017.01750 29321777 PMC5732229

[40] Eelen G de Zeeuw P Treps L Harjes U Wong BW Carmeliet P. Endothelial cell metabolism. *Physiol Rev.* (2018) 98:3–58. 10.1152/physrev.00001.2017 29167330 PMC5866357

[41] Fu L Liu K He J Tian C Yu X Yang J. Direct proteomic mapping of cysteine persulfidation. *Antioxid Redox Signal.* (2019) 2019:7777. 10.1089/ars.2019.7777 31411056

[42] Marutani E Yamada M Ida T Tokuda K Ikeda K Kai S Thiosulfate mediates cytoprotective effects of hydrogen sulfide against neuronal ischemia. *J Am Heart Assoc.* (2015) 4:11. 10.1161/JAHA.115.002125 26546573 PMC4845224

[43] Lee M McGeer EG McGeer PL. Sodium thiosulfate attenuates glial-mediated neuroinflammation in degenerative neurological diseases. *J Neuroinflam.* (2016) 13:32. 10.1186/s12974-016-0488-8 26856696 PMC4746933

[44] Kolluru GK Shen X Bir SC Kevil CG. Hydrogen sulfide chemical biology: pathophysiological roles and detection. *Nitric Oxide.* (2013) 35:5–20. 10.1016/j.niox.2013.07.002 23850632 PMC4077051

[45] Katsouda A Bibli SI Pyriochou A Szabo C Papapetropoulos A. Regulation and role of endogenously produced hydrogen sulfide in angiogenesis. *Pharmacol Res.* (2016) 113:175–85. 10.1016/j.phrs.2016.08.026 27569706 PMC5107115

[46] Cai WJ Wang MJ Moore PK Jin HM Yao T Zhu YC. The novel proangiogenic effect of hydrogen sulfide is dependent on Akt phosphorylation. *Cardiovasc Res.* (2007) 76:29–40. 10.1016/j.cardiores.2007.05.026 17631873

[47] Kolluru GK Bir SC Yuan S Shen X Pardue S Wang R Cystathionine gamma-lyase regulates arteriogenesis through NO-dependent monocyte recruitment. *Cardiovasc Res.* (2015) 107:590–600. 10.1093/cvr/cvv198 26194202 PMC4540149

[48] Potenza DM Guerra G Avanzato D Poletto V Pareek S Guido D Hydrogen sulphide triggers VEGF-induced intracellular Ca(2)(+) signals in human endothelial cells but not in their immature progenitors. *Cell Calcium.* (2014) 56:225–34. 10.1016/j.ceca.2014.07.010 25113159

[49] Bir SC Kolluru GK McCarthy P Shen X Pardue S Pattillo CB Hydrogen sulfide stimulates ischemic vascular remodeling through nitric oxide synthase and nitrite reduction activity regulating hypoxia-inducible factor-1alpha and vascular endothelial growth factor-dependent angiogenesis. *J Am Heart Assoc.* (2012) 1:e004093. 10.1161/JAHA.112.004093 23316304 PMC3541625

[50] Fung E Helisch A. Macrophages in collateral arteriogenesis. *Front Physiol.* (2012) 3:353. 10.3389/fphys.2012.00353 23055975 PMC3457069

[51] Grundmann S Piek JJ Pasterkamp G Hoefer IE. Arteriogenesis: basic mechanisms and therapeutic stimulation. *Eur J Clin Invest.* (2007) 37:755–66. 10.1111/j.1365-2362.2007.01861.x 17764463

[52] Zhang J Muri J Fitzgerald G Gorski T Gianni-Barrera R Masschelein E Endothelial lactate controls muscle regeneration from ischemia by inducing M2-like macrophage polarization. *Cell Metab.* (2020) 31:1136–53.e7. 10.1016/j.cmet.2020.05.004 32492393 PMC7267778

[53] Naito Y Takagi T Higashimura Y. Heme oxygenase-1 and anti-inflammatory M2 macrophages. *Arch Biochem Biophys.* (2014) 564:83–8. 10.1016/j.abb.2014.09.005 25241054

[54] Pan LL Qin M Liu XH Zhu YZ. The role of hydrogen sulfide on cardiovascular homeostasis: an overview with update on immunomodulation. *Front Pharmacol.* (2017) 8:686. 10.3389/fphar.2017.00686 29018349 PMC5622958

[55] Wu J Chen A Zhou Y Zheng S Yang Y An Y Novel H2S-releasing hydrogel for wound repair via in situ polarization of M2 macrophages. *Biomaterials.* (2019) 222:119398. 10.1016/j.biomaterials.2019.119398 31487582

