# Supplementary material for: Sodium thiosulfate, a source of hydrogen sulfide, stimulates endothelial cell proliferation and neovascularization
Source: Front Cardiovasc Med. 2022 Oct 3;9:965965. doi: 10.3389/fcvm.2022.965965 (PMC9575962; doi:10.3389/fcvm.2022.965965)
Supplement: Supplementary file 1 [file Data_Sheet_1.docx]

**Supplementary Data**

**Sodium Thiosulfate, a source of Hydrogen Sulfide, stimulates endothelial cell proliferation and neovascularization.**

Diane Macabrey^1^, Jaroslava Joniová^2^, Quentin Gasser^1^, Clémence Bechelli^1^, Alban Longchamp^1^, Severine Urfer^1^, Martine Lambelet^1^, Chun-Yu Fu^3^, Guenter Schwarz^3^, Georges Wagnieres^2^, Sébastien Déglise^1*^ and Florent Allagnat^1*^

*^1^Department of Vascular Surgery, Lausanne University Hospital, Switzerland*

*^2^Laboratory for functional and metabolic imaging, LIFMET, Swiss Federal Institute of Technology (EPFL), Lausanne, Switzerland.*

*^3^Institute of Biochemistry, Department of Chemistry, University of Cologne, Germany*

*These authors contributed equally to this work.

Corresponding Author :

Florent Allagnat

CHUV-Service de chirurgie vasculaire

Département des Sciences Biomédicales

Bugnon 7A, 1005 Lausanne, Suisse

[Florent.allagnat@chuv.ch](mailto:Florent.allagnat@chuv.ch)

Running title: sodium thiosulfate promotes angiogenesis

Word count: 7550

6 Figures

Category: original article

Keywords: angiogenesis; endothelial cells, proliferation; hydrogen sulfide; sodium thiosulfate, arteriogenesis, endothelial cell metabolism, reperfusion, peripheral arterial disease.

## **Supplemental Method**

## Western blotting

Ischemic and contralateral gastrocnemius muscles were collected and flash-frozen in liquid nitrogen, grinded to power and resuspended in SDS lysis buffer (62.5 mM TRIS pH6,8, 5% SDS, 10 mM EDTA). Protein concentration was determined by DC protein assay (Bio-Rad Laboratories, Reinach, Switzerland). 10 to 20 µg of protein were loaded per well, as previously described^22, 26^. Lysates were resolved by SDS-PAGE and transferred to a PVDF membrane (Immobilon-P, Millipore AG, Switzerland). Immunoblot analyses were performed as previously described^26^ using the antibodies described in **Supplemental Table 1.** Membranes were revealed by enhanced chemiluminescence (Immobilon, Millipore) using the Azure 280 device (Azure Biosystems) and analysed using Fiji (ImageJ 1.53c). Protein abundance was normalised to total protein using Pierce™ Reversible Protein Stain Kit for PVDF Membranes (cat 24585; Thermo Fisher Scientific)*.*

**Supplemental Figures**

**
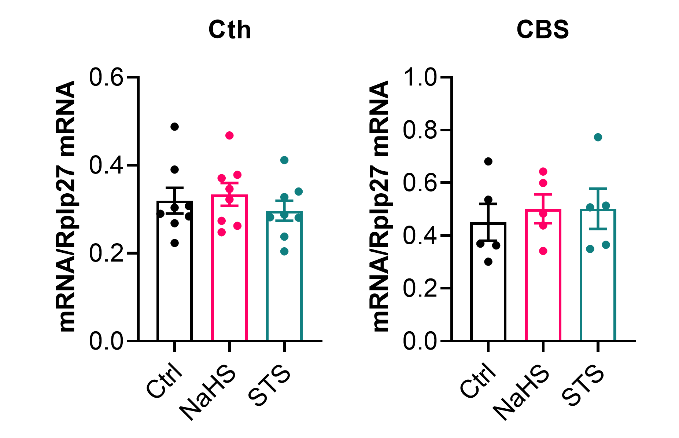
**

**Figure S1. STS does not affect Cth or Cbs gene expression**

Cth and Cbs mRNA expression in HUVEC exposed for 4h to 100µM NaHS or 3mM STS. Data are mean ± SEM. No statistical differences as assessed by paired One-way ANOVA with DUNNET’s correction for multiple comparisons.

**
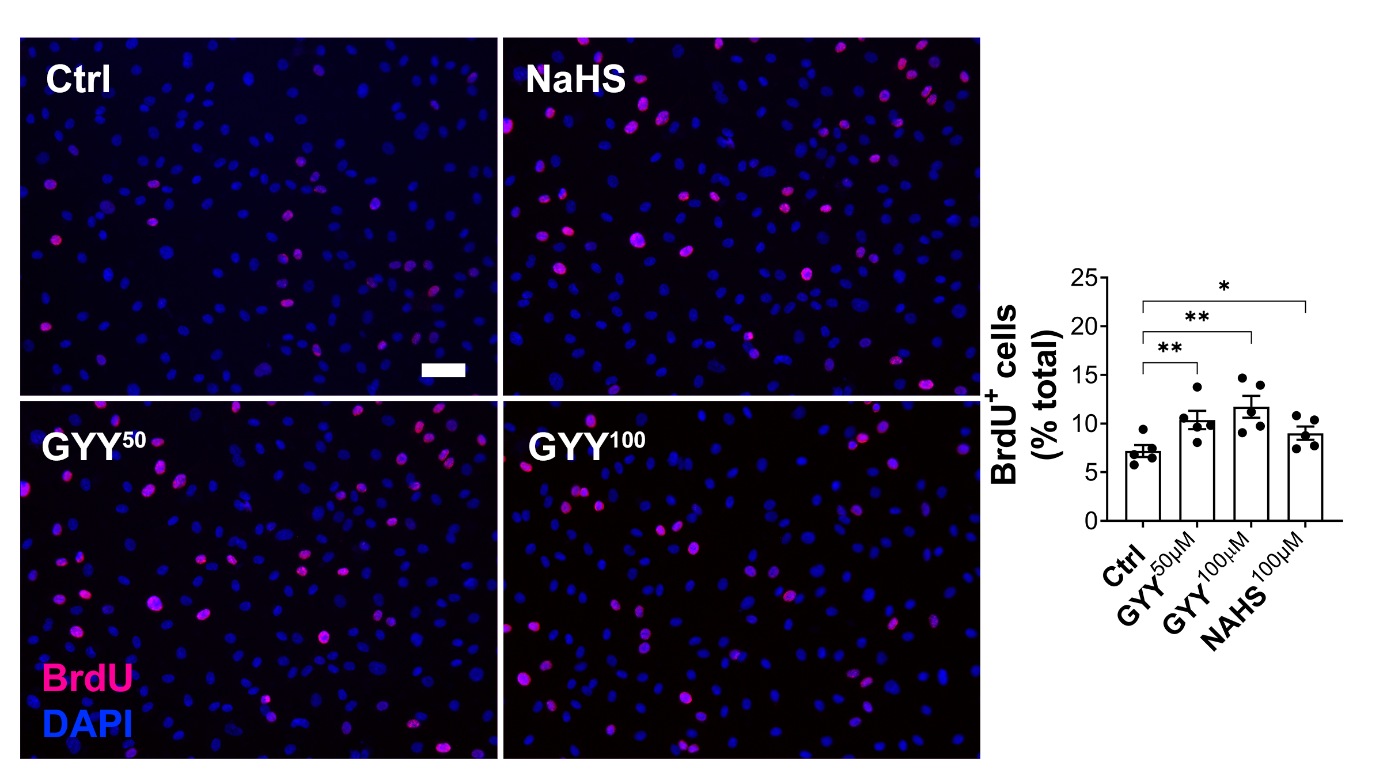
**

**Figure S2. H_2_S donors stimulate HUVEC proliferation**

HUVEC proliferation was assessed by BrdU incorporation and expressed as BrdU positive cells (pink) over DAPI positive nuclei. Data shown as mean ± SEM of 5 independent experiments. *p<.05 **p<.01 ***p<.001 as determined by paired one-way ANOVA with Dunnett’s post-hoc test.

**
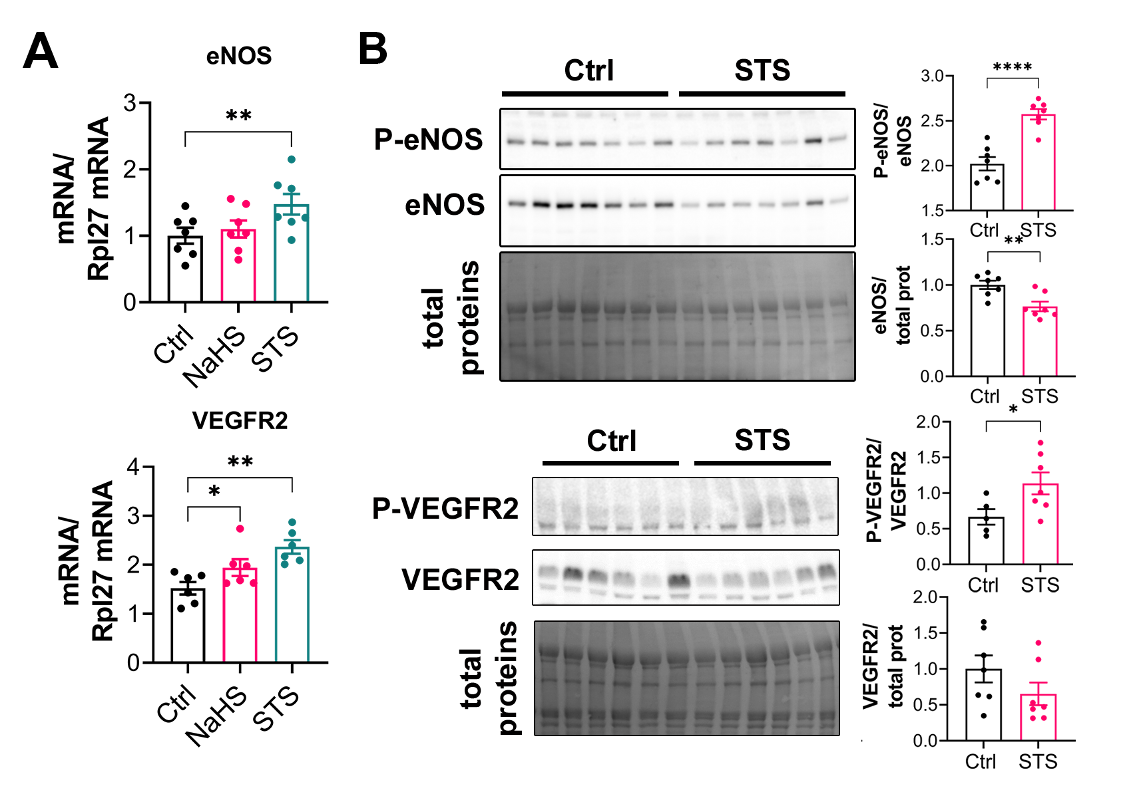
**

**Figure S3. STS promotes VEGFR2 and eNOS phosphorylation**

**A**) Normalized eNOS and VEGFR2 mRNA levels in HUVEC treated or not (Ctrl) with 100µM NaHS or 15mM STS for 4h. *p<.05, **p<.01 as determined by paired one-way ANOVA with Dunnett’s post-hoc tests. **B**) Western blot on ischemic muscles after 14 days of STS treatment, P-eNOS levels normalized with total eNOS levels, VEGFR2 levels normalized to total proteins. *p<.05, **p<.01, ****p<.0001 as determined by bilateral unpaired t-test. All proteins normalized to total protein stain.


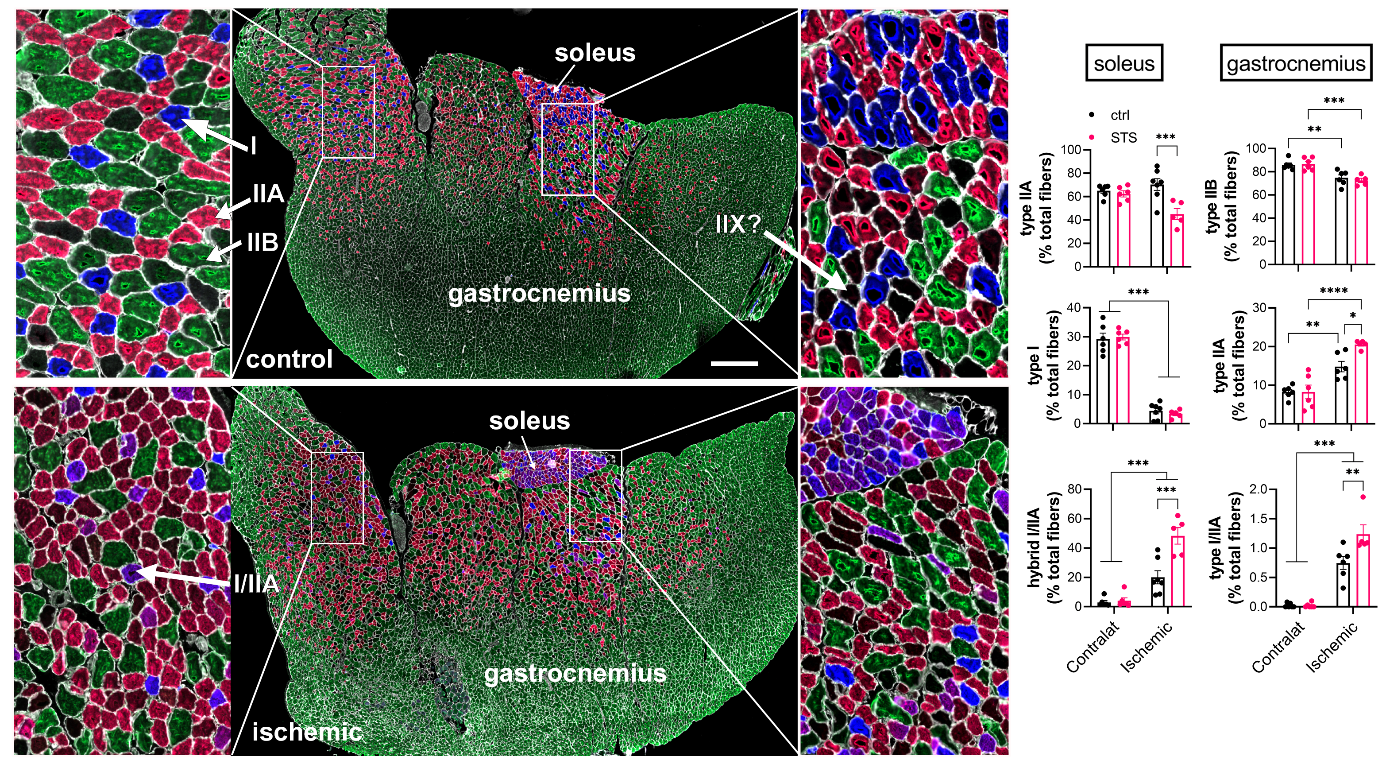


**Figure S4: ischemia induces a shift from slow to fast fiber types in the gastrocnemius and soleus muscle**

Representative cross section of the gastrocnemius and soleus muscle of contralateral leg and ischemic leg of WT mice 14 days post ischemic injury. Muscle fibers are stained to delineate individual type I slow (Myh7 in blue), type IIA fast (Myh2 in red) oxidative fibers, and type IIB fast glycolytic fibers (Myh4 in green). Basement membrane is stained with laminin (white). Quantitative assessment of the fiber types after images analysis in ImageJ. Data are ± SEM of 5 to 7 animals per group.*p<.05; **p<.01;***p<.001 by two-way ANOVA with Sidak’s multiple comparisons tests.

**Supplementary Table S1: Antibodies**

| **Target antigen** | **Vendor** | **Catalog #** | **Working**  **concentration** |
| --- | --- | --- | --- |
| **Laminin** | Sigma | L9393 | 1/200 (IHC) |
| **Ve-Cadherin** | Abcam | AB33168 | 1/100 (IHC) |
| **CD68** | Biorad | MCA1957T | 1/500 (IHC) |
| **HO-1** | Abcam | 13243 | 1/200 (IHC) |
| **ERG** | CST | #4695 | 1/100 (IHC) |
| **Anti-Rabbit HRPO** | Thermo Fisher Scientific | 31460A21109 | 1/20000 (WB) |
| **Anti-mouse HRPO** | Jackson ImmunoResearch Labs | 115-035-146 | 1/15000 (WB) |
| **Anti-Rabbit HRPO** | Thermo Fisher Scientific | 31460 | 1/20000 (WB) |
| **BrdU** | BD Biosciences | 555627 | 1/200 (ICC) |
| **eNOS** | BD Biosciences | 610297 | 1/1000 (WB) |
| **peNOS** | CST | #9571 | 1/500 (WB) |
| **VEGFR2** | CST | #2479 | 1/1000 (WB) |
| **pVEGFR2** | CST | #2478 | 1/500 (WB) |
| **SMA** | CST | #19245 | 1/500 (IHC) |
| **Myh7** | Developmental Studies Hybridoma Bank | BA-D5 (MIgG2b) | 1:50 in PBS 2% goat serum (IHC) |
| **Myh2** | Developmental Studies Hybridoma Bank | SC-71 (MIgG1) | 1:50 in PBS 2% goat serum (IHC) |
| **Myh4** | Developmental Studies Hybridoma Bank | BF-F3 (MIgM) | 1:50 in PBS 2% goat serum (IHC) |
| **Goat anti-Rabbit Alexa Fluor 680** | Thermo Fisher Scientific | A21109 | 1/500 (IHC) |
| **Goat anti-Rabbit Alexa Fluor 405** | Thermo Fisher Scientific | A31556 | 1/500 (IHC) |
| **Goat anti-Rat Alexa Fluor 488** | Thermo Fisher Scientific | A11006 | 1/500 (IHC) |
| **Donkey anti-Rabbit Alexa Fluor 488** | Thermo Fisher Scientific | A21206 | 1/500 (IHC) |

**Supplemental table S2: DNA oligo primers**

| **Primer Name** | **Sequence** |
| --- | --- |
| **hTSTD1** | Fw: TCAACATCCCGGTGTCCGAG  Rv: TCCAGCTTTGGCTTCTCAGC |
| **hSUOX** | Fw: GGTGCAGTGTTGGCCTATCA  Rv: ACCCAGATCCCAGTCTCAGG |
| **hMPST** | Fw: CCGAGACGGCATTGAACCT  Rv: CCTGGCTCAGGAAGTCTGTG |
| **hTST** | Fw: GGAGCCGGATATAGTAGGACT  Rv: AATATGGCACGCAGCTCCTC |
| **hSQOR** | Fw: GACGAGAAGATTCTCCTACCGA  Rv: TTGGGATGAGCGAAACCTTCA |
| **hETHE1** | Fw: GCATGGCCTTCACTGGAGAT  Rv: TGGACCGAGTGGTACAAGGT |
| **hCTH** | Fw: CCAGCACTCGGGTTTTGAAT  Rv: TACTTAGCCCCATCCAGTGC |
| **hCBS** | Fw: ATGGTGACGCTTGGGAACAT  Rv: GGCGGATCTGTTTGAACTGC |
| **hNOS3** | Fw:GCCGGAACAGCACAAGAGTTA  Rv:CCCTGCACTGTCTGTGTTACT |
| **hVEGFR2** | Fw: CCCAGGCTCAGCATACAAAAAG  Rv: CTGCACTCAGTCACCTCCAC |
| **mSuox** | Fw: CATCGGTGTAGGGCTTCTCA  Rv: AGGGTTGTTGTGAGAACGCA |
| **mMpst** | Fw:GGCCACCACTCTGTGTCATT  Rv:GGAGCTGATTGGCAGGTTCT |
| **mTst** | Fw: GGAGCCGGATATAGTAGGACT  Rv: AATATGGCACGCAGCTCCTC |
| **mSqor** | Fw:TGGAATATGATGCTCAGAGGCT  Rv:AAAGCAAGCTGTGAGCCATC |
| **mEthe1** | Fw; ACAGACTTCCAACAAGGCTGT  Rv:TGGTAATCGTGAGCCGGGT |
| **mPfkfb3** | Fw: AAATGTCCGCTCCACACTGT  Rv: GGTGTGTGCTCACCGATTCT |
| **mPKM** | Fw: TCGCATGCAGCACCTGATAG  Rv: TCCATGAGGTCTGTGGAGTGA |
| **mTstd2** | Fw: CCTAGGGTTTGCGGATACTTG  Rv: TCGTCTGGTGAAGTGGAAGAAG |
